# Chemotaxis Towards Aromatic Compounds: Insights from *Comamonas testosteroni*

**DOI:** 10.3390/ijms20112701

**Published:** 2019-06-01

**Authors:** Yun-Hao Wang, Zhou Huang, Shuang-Jiang Liu

**Affiliations:** 1 State Key Laboratory of Microbial Resources and Environmental Microbiology Research Center, Institute of Microbiology, Chinese Academy of Sciences, Beijing 100101, China; wangyunhao_123@yeah.net (Y.-H.W.); huangzhou_129@msn.com (Z.H.); 2 University of Chinese Academy of Sciences, Beijing 101408, China

**Keywords:** *Comamonas testosteroni*, chemoreceptor, chemotaxis, aromatic compounds

## Abstract

Chemotaxis is an important physiological adaptation that allows many motile bacteria to orientate themselves for better niche adaptation. Chemotaxis is best understood in *Escherichia coli*. Other representative bacteria, such as *Rhodobacter sphaeroides*, *Pseudomonas* species, *Helicobacter pylori*, and *Bacillus subtilis*, also have been deeply studied and systemically summarized. These bacteria belong to α-, γ-, ε-Proteobacteria, or Firmicutes. However, β-Proteobacteria, of which many members have been identified as holding chemotactic pathways, lack a summary of chemotaxis. *Comamonas testosteroni*, belonging to β-Proteobacteria, grows with and chemotactically responds to a range of aromatic compounds. This paper summarizes the latest research on chemotaxis towards aromatic compounds, mainly from investigations of *C. testosteroni* and other *Comamonas* species.

## 1. Introduction

Aromatic compounds have one or more aromatic rings containing resonance bonds, which make them chemically inert due to charge distribution over the whole skeleton of the aromatic ring. It has been estimated that about 25% of photosynthetic products from plants are deposited as lignin, which is one of the most abundant aromatic compounds on earth [1,2]. Other biogenic aromatic compounds, such as phenylalanine, tyrosine, tryptophan, many hormones, and signal molecules, play important roles in the maintenance and regulation of biological processes. Chemically synthesized aromatic compounds are important raw materials. Benzene, phenol, and toluene are classified as priority pollutants [3]. The toxicity and mutagenicity of nitroaromatic compounds have been documented [4,5]. 

Microbial biodegradation is an important process for removal of aromatic pollutants from environments [6,7]. In addition to metabolic robustness, microbial degradation also relies on the bioavailability of aromatic pollutants. Bacterial chemotactic responses toward aromatic compounds have attracted extensive interest [8,9,10] and are beneficial for bacteria to find more friendly environments and to degrade pollutants [11,12,13]. Chemotaxis may also facilitate the transfer of catabolic genes via motile bacteria to contaminated environments [14], thus promoting the coexistence of biodegradation and chemotaxis genes. Reviews are available on bacterial chemotaxis regarding stimulus-sensing and signal-processing [15], on exploring the function of bacterial chemotaxis [16], and on *Pseudomonas* chemotaxis [17]. This review is focused on the recent progress made on the study of bacterial chemotaxis towards aromatic compounds, in particular with *Comamonas testosteroni*.

## 2. The Taxonomy, Ecology, Physiology, and Chemotaxis of *C. testosteroni*

**Figure 1:**
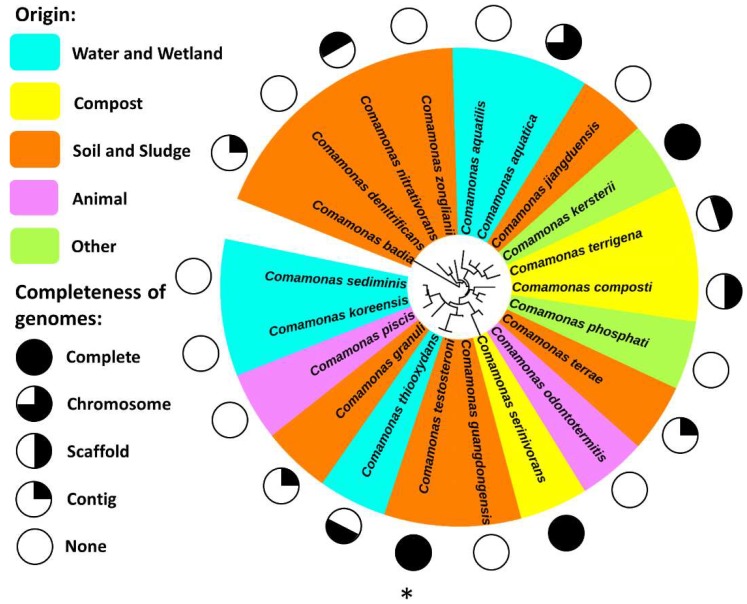
The genus *Comamonas*: phylogeny, diversity, habitats, and genomes. The phylogenetic analysis is based on 16S rRNA genes. Background colors represent the origins of type strains of species. The completeness of genomes is indicated by circles outside the species names. *Comamonas testosteroni* is indicated by an asterisk (*).

*C. testosteroni* belongs to the genus *Comamonas* that has 21 species (http://www.bacterio.net/comamonas.html) (Figure 1). The *Comamonas* species are Gram-negative bacteria belong to β-Proteobacteria, and are distributed widely in soil, sediments, and garden ponds [18,19,20]. Based on the IMG database (https://img.jgi.doe.gov/), 11 of the 21 *Comamonas* species have been genome-sequenced [21], and mining these genomes indicated that the key genes for hexose phosphorylation were missing [22]. Thus, unlike *Pseudomonas* species that prefer to assimilate carbohydrates for growth, the *Comamonas* species do not use glucose as a sole carbon source, but grow well with non-sugar carbon sources such as organic acids and aromatic compounds. Although *Comamonas* and *Pseudomonas* species are different in carbohydrate assimilation, they share similar ecological habitats, and both are able to metabolize aromatic compounds. Studies have demonstrated that *Comamonas* species play an important role in the biological geocycling of nutrients in environments [23,24,25], as well as in bioremediation of polluted environments [26,27,28]. A search on Web of Science using the key word “*Comamonas*” revealed more than 1700 publications from 2000 to 2018, and citation of these publication has increased sharply in recent years, which suggests a growing interest from scientists. The *C. testosteroni* strain CNB-1 was isolated from activated sludge and grows using chloronitrobenzene as carbon and nitrogen sources [29]; it has been exploited for the bioremediation of chloronitrobenzene-polluted soil [30]. The genes involved in the degradation of chloronitrobeneze were located on a mega-plasmid pCNB1 [31], and the degradation pathway was identified [32]. Another strain of *C. testosteroni*, CNB-2, is a derivative from strain CNB-1 from which pCNB1 was cured [33].

Bacterial chemotaxis is the movement of cells with either flagella, pili, or gliding structures in response to chemical gradients. The well-studied enteric *Escherichia coli* has only one gene cluster and five chemoreceptors for chemotaxis. However, many environmental bacteria have more gene clusters and chemoreceptors. Studies show that the number of chemoreceptors in genomes is relevant to lifestyles, but not to genome sizes [34]. *C. testosteroni* strain CNB-1 is motile with 1–3 polar flagella and chemotactically responds to a range of aromatic and other organic compounds (Table 1). In contrast to *E. coli*, data-mining of the CNB-1 strain genome revealed two chemotaxis-like gene clusters and 19 putative chemoreceptor genes.

## 3. Chemoreceptors of *C. testosteroni*

According to the well-studied enteric bacteria *E. coli* and *Salmonella enterica*, typical chemotactic responses start with signals generated by transmembrane chemoreceptors, also called methyl-accepting chemotaxis proteins (MCPs). Upon conformational changes of chemoreceptors, the phosphotransfer starts from histidine kinase CheA to response regulator CheY [15]. Phosphorylated CheY interacts with the flagellar switch protein FliM and, as a result, reverses flagellar rotation [40]. The chemotactic proteins phosphatase CheZ [41], methyltransferase CheR [42], and methylesterase CheB [43] contribute to signal termination and adaptation. *C. testosteroni* has a dozen predicted chemoreceptors, one histidine kinase, two response regulators, and also has phosphorylation and methylation systems.

### 3.1. Genomic Data-mining of Chemoreceptor Genes

Analysis of the *C. testosteroni* CNB-1 genome using the MiST.2 database [44] revealed 19 chemoreceptor genes. The putative chemoreceptor genes of other *Comamonas* genomes were also explored. For example, *C. kerstersii* has 25 and *C. aquatic* 35 chemoreceptor genes. Surprisingly, *C. serinivorans* has only one chemoreceptor gene on its entire genome. It would be interesting to know how this bacterium senses and responds to different environmental signals in the environment.

### 3.2. Classification of Chemoreceptors from C. testosteroni CNB-1

**Figure 2:**
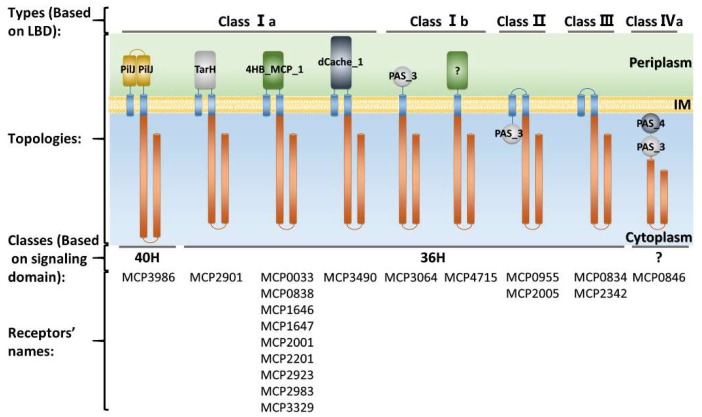
The chemoreceptor repertoires of *C. testosteroni* CNB-1 and their classification. Domain architecture of chemoreceptors was identified by using CDvist [49].

Based on signaling domain sequences in chemoreceptors, the predicted nineteen chemoreceptors of strain CNB-1 belong to two major classes, 40H and 36H (Figure 2) [45]. Seventeen of the nineteen chemoreceptors are assigned to the 36H class. Members of the 36H class chemoreceptors are known to interact with the chemotaxis signal transduction class F7 [46,47]. The 36H chemoreceptors MCP2201, MCP2983, and MCP2901 from *C. testosteroni* CNB-1 were experimentally shown to be involved in chemotaxis towards di- and tricarboxylic acids and aromatic compounds [36,37,38]. The only 40H class chemoreceptor MCP3986 has recently been identified to be involved in biofilm formation [48]. The last chemoreceptor, MCP0846, does not match confidently to any classes (Figure 2).

Besides the signaling domain, the ligand-binding domain (LBD) that senses environmental stimuli is also used for chemoreceptor classification. Wuichet et al. [50] classified chemoreceptors according to their topologies into four classes (I–IV). Lacal et al. [51] further distinguished class I as two subclasses, according to the number of transmembrane (TM) regions of chemoreceptors. The class IV chemoreceptors can also be divided into two subclasses according to the presence or the absence of LBDs [51]. As shown in Figure 2, the most abundant topology in *C. testosteroni* CNB-1 is class I chemoreceptors that harbor pericytoplasmic LBDs: 12 chemoreceptors belong to class Ia and two chemoreceptors belong to class 1b. MCP2005 and MCP0955, both containing a cytoplasmic PAS domain, belong to class II topology. MCP0834 and MCP2342 have one or two TM regions but no LBDs, so they can be assigned to class III topology. Their functions in chemotactic sensory are still unknown. MCP0846 has a class IVa topology with two PAS domains and no TM helices (Figure 2).

## 4. Functional Identification of Chemoreceptors from *C. testosteroni*

### 4.1. Bioinformatic Prediction of Potential Ligand-Binding Domains

The LBD sequences are more divergent compared with the signaling domains of chemoreceptors. According to Ortega et al. [52], more than 80 different LBD types were found in the Pfam 31.0 database. As shown in Figure 2, there are 10 chemoreceptors equipped with LBDs composed of 4-helix bundles (4HB_MCP_1 and TarH) in CNB-1. There are four chemoreceptors containing PAS domains: MCP0846, MCP0955, MCP2005, and MCP3064 (Figure 2). The chemoreceptors MCP3490 and MCP3986 contain dCache and PilJ domains, respectively (Figure 2). The LBD of MCP4715 is an unknown LBD type (Figure 2). There is no LBD identified in MCP0834 and MCP2342 (Figure 2), which raised the question as to how they sense stimulating signals. The diversity of LBDs is the structural basis for chemoreceptors to recognize diverse environmental signals. Meanwhile, this diversity also resulted in the complexity and difficulty of LBDs functional annotation, although a variety of ways to annotate them have been introduced [53].

### 4.2. Functional Redundancy of Chemoreceptors

Recent studies have shown functional redundancy of chemoreceptors in *P. aeruginosa* [54] and other bacteria [55,56]. In *C. testosteroni* CNB-1, the 19 putative chemoreceptors were individually disrupted, and the resulting mutants were tested for chemotaxis towards aromatic and other organic compounds with semisoft agar plate assays. Results showed that none of these mutants completely lost chemotactic responses to the tested compounds, although several mutants showed weakened chemotactic responses. These observations indicated that the chemoreceptors in *C. testosteroni* were likely to be functionally overlapping or redundant. A chemoreceptor-null mutant CNB-1Δ20 was created by deletion of all 19 chemoreceptor genes (this mutant was named CNB-1Δ20 because the gene MCP2901 was deleted two times). As expected, this mutant CNB-1Δ20 completely lost its chemotactic responses. With this chemoreceptor-null mutant CNB-1Δ20, we were able to identify the functional redundancy of chemoreceptors in *C. testosteroni* via complementation of individual chemoreceptor genes. All three chemoreceptors MCP2201, MCP2901, and MCP2983 were able to trigger metabolism-dependent chemotaxis towards aromatic compounds, although they have different ligand-binding spectra: MCP2201 binds to oxaloacetate, citrate, *cis*-aconitate, isocitrate, α-ketoglutarate, succinate, fumarate, and malate [36]; MCP2983 only binds to *cis*-aconitate [37]; and MCP2901 binds to citrate, as well as to aromatic molecules such as gentisate, 4-hydroxybenzoate, 2,6-dihydroxybenzoate, and 2-hydroxybenzoate [38]. The advent of the MCP-null mutant CNB-1Δ20 is critical to overcome functional redundancy of chemoreceptors, and this mutant CNB-1Δ20 can be used to identify chemoreceptors from other bacteria.

## 5. Chemotaxis of *C. testosteroni* Towards Aromatic Compounds

**Figure 3:**
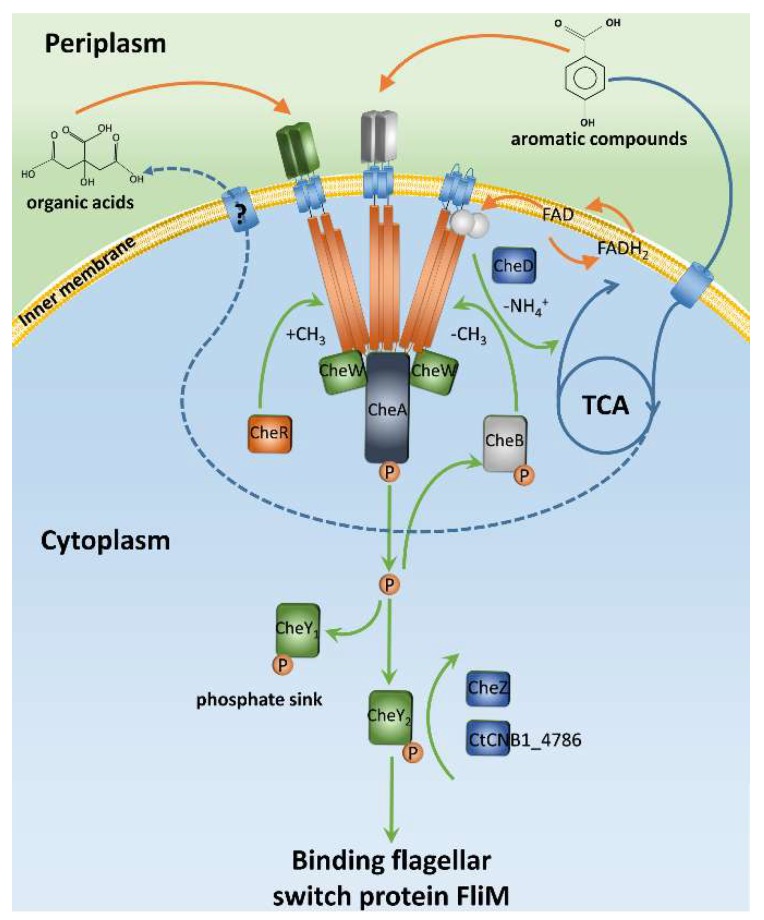
The *Comamonas testosteroni* CNB-1 chemotaxis pathway. Both metabolism-dependent and -independent chemotaxis exist in CNB-1 simultaneously. Some chemoreceptors directly sense aromatic compounds, and others sense metabolic intermediates or energy levels. The dotted line indicates a postulate pathway of material transportation.

Chemotaxis towards aromatic compounds has been widely documented for various bacteria, including members of *Pseudomonas* [9,14], *Acidovorax* [35], *Ralstonia* [57,58], *Agrobacterium* [59], *Azospirillum* [60], *Bradyrhizobium*, and *Rhizobium* [61]. *C. testosteroni* grows with a variety of aromatic compounds, and many of these compounds also serve as chemotactic attractants. Both metabolism-dependent and metabolism-independent chemotaxis, as defined by Pandey and Jain [62], have been reported for *C. testosteroni* [36,38]. The well-known metabolism-dependent chemotaxis of aromatic compounds is Aer-like chemoreceptors mediated chemotaxis. The PAS domains of Aer-like chemoreceptors are able to sense the concentrations of FAD molecules during metabolism of compounds. It was demonstrated that Aer2 [63] and three other polar localized Aer-like proteins [64] triggered chemotaxis of *P. putida* towards (methyl)phenols and phenylacetic acid. *Acidovorax* species also uses an Aer chemoreceptor for taxis towards 2-nitrotoluene [35]. *C. testosteroni* CNB-1 has four Aer-like chemoreceptors (MCP0846, MCP0955, MCP2005, and MCP3064) with PAS domains, which have tryptophan as a specific recognition site of FAD [65]. MCP0955, MCP2005, and MCP3064 have typical transmembrane regions and are predicted to be membrane-located, while MCP0846 does not have transmembrane regions and is predicted to be a cytoplasmic chemoreceptor. Although the functions of these chemoreceptors have not been experimentally identified, their functions presumably are the same as other Aer-like chemoreceptors (Figure 3).

Besides the Aer-like chemoreceptors that sense FAD molecule, recent studies have demonstrated that other chemoreceptors trigger chemotaxis towards aromatic compounds via sensing intermediate metabolites [36,37,38]. The chemoreceptors MCP2201 and MCP2983 from *C. testosteroni* mediated chemotaxis towards aromatic compounds via binding to citrate, cis-aconitate, succinate, and other molecules of Kreb’s cycle when grows with aromatic compounds (Figure 3) [36,37]. Chemoreceptors that bind to and sense Kreb’s cycle intermediates were reported in other bacteria such as *P. aeruginosa*, *P. putida*, and *Salmonella typhimurium* [66,67,68]. One of these chemoreceptors, TcpS, was able to trigger chemotaxis towards aromatic compounds when it was complemented into *C. testosteroni* Δ20 [36]. Evolution of metabolism-dependent chemotaxis towards aromatic compounds may occur widely in microbes, and it is a shrewd strategy. This strategy converges a wide variety of substances into a few metabolic intermediates (including FAD) as chemoeffectors, so it would reduce the variety of chemoreceptors and save energy. Metabolism-independent chemotaxis was also reported, and chemoreceptors that were putatively binding directly to aromatic compounds were proposed. The chemoreceptor NahY from *P. putida* triggered chemotaxis towards naphthalene [69,70], and the chemoreceptor NbaY of *P. fluorescens* triggered chemotaxis towards 2-nitrobenzoate [71]. Moreover, the McpT of *P. putida* DOT-T1E and CtpL of *P. aeruginosa* PAO1 reportedly trigger chemotactic responses to toluene, 4-chloroaniline, and catechol [72,73]. However, the ligand-binding properties of those chemoreceptors were not demonstrated. Recently, studies reported that the McpP from *P. putida* bound C2 and C3 carboxylic acids, which had been proposed being a chemoreceptor for benzoate. McpP is homologeous to NbaY, and investigation showed that neither McpP nor NbaY bound to benzoate, nitrobenzoate, or other substituted benzoates [74]. The first biochemical evidence that demonstrated a chemoreceptor directly bound to aromatic compounds was from *C. testosteroni*: MCP2901 binds to 2,6-dihydroxybenzoate and 2-hydroxybenzoate and triggers signal transduction for chemotaxis (Figure 3) [38]. So far, the other known chemoreceptor that binds to aromatic compounds is the PcaY for protocatechuate from *P. putida* KT2440 [75]. We have also biochemically characterized PcaY from *P. putida* F1, and its ligand is also protocatechuate. Compared to metabolism-dependent chemotaxis, metabolism-independent chemotaxis enables microbes to have a faster response and improves the chances of survival. For some non-metabolizable substances such as toxic compounds and metal, metabolism-independent chemotaxis is the only choice.

## 6. Chemotactic Signaling Pathways in *C. testosteroni* and Other *Comamonas* Species

**Figure 4:**
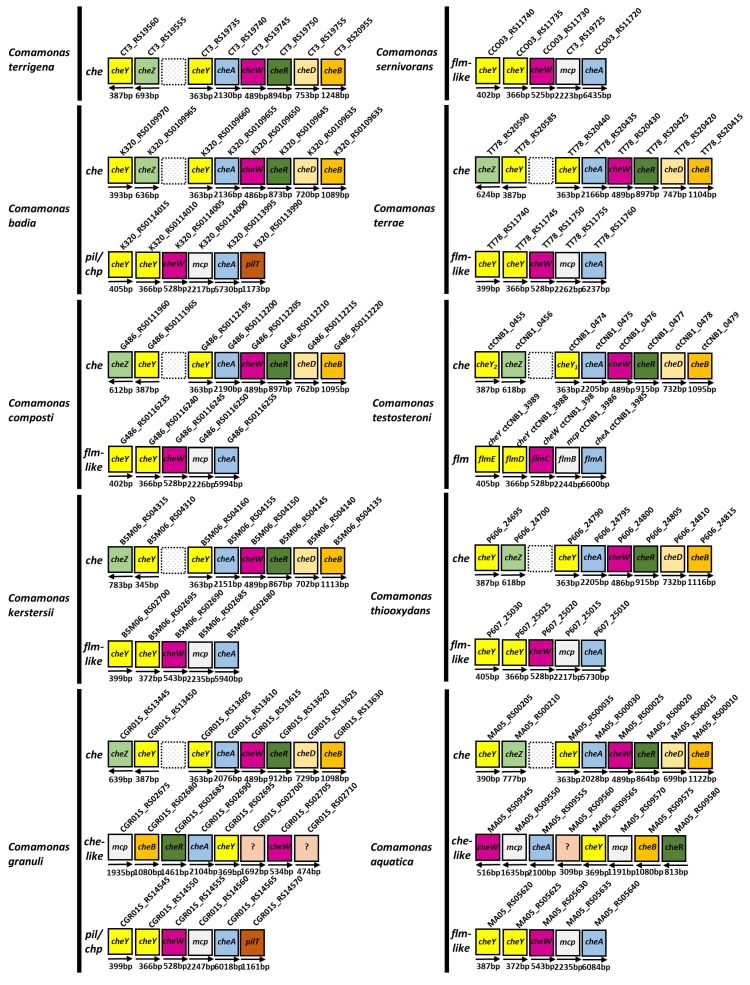
Data-mining of *che* and *che-like* gene clusters in *C. testosteroni* and other *Comamonas* genomes. There are in total 11 *Comamonas* genomes in publically available databases. Except for *C. nitrativorans* (IMG Genome ID: 2574180442), in which there was no *che* or *che-like* gene cluster, the *che* and *che-like* gene clusters of the other 10 *Comamonas* genomes were shown. GenbBank IDs are BCNR01000016.1 (*C. terrigena*); CP021455.1 (*C. serinivorans*); AXVM00000000.1 (*C. badia*); BCNT01000000.1 (*C. terrae*); AUCQ01000000.1 (*C. composti*); CP001220.2 (*C. testosteroni*); CP020121.1 (*C. kerstersii*); AWTO01000000.1 (*C. thiooxydans*); BBJX01000000.1 (*C. granuli*); and CP016603.1 (*C. aquatica*). The lengths of rectangles are independent of the lengths of gene sequences.

So far, the chemosensory signaling pathway in enteric *E. coli* has been extensively characterized [76,77]. However, environmental bacteria often have more complex chemotactic signaling systems [15,78] than does *E. coli*. Based on genome data-mining, half of chemotactic bacteria of which genomes have been sequenced have more than one CheAs [46]. Besides phosphatases CheZ, CheC, FliY, and CheX also have dephosphorylation activities for CheY-P [79]. In addition, other chemotactic proteins have been found: CheD has deamination activity for key glutamine residues of chemoreceptors. CheV, a CheW-like coupling protein, is a scaffold between CheA and chemoreceptors [80]. These genetic organizations of chemotactic genes in environmental bacteria are more complex than in enteric *E. coli*. Bacteria such as *R. sphaeroides*, *B. subtilis*, and *P. aeruginosa* have additional gene clusters, and those clusters are similar to the *che* cluster [81]. Most of these additional clusters are involved in modulation of chemotaxis [82,83], but some of them are involved in the control of alternative biological behavior, including twitching motility [84], biofilm formation [85], cell development [86], and flagellar biosynthesis [87]. In *C. testosteroni* CNB-1, there are *che* and *che-like* clusters, respectively (Figure 4). The *che* cluster contains a complete set of chemotaxis genes, and is essential for chemotaxis. Compared to that of *E. coli*, the *che* cluster of *C. testosteroni* CNB-1 has two CheYs. The CheY_2_ is FliM-binding and is the true chemotaxis response regulator in CNB-1. The function of CheY_1_ is a phosphate sink [48]. In addition to the phosphatase CheZ on the *che* cluster, we have recently identified an additional gene (CtCNB_4786) that showed low sequence identity to CheZ but is involved in chemotactic signal quenching (unpublished data). The *che-like* gene cluster regulates biofilm formation, and was recently named *flm* cluster. Cross-talk between *che* and *flm* clusters via phosphotransfer was identified, and coordination of chemotaxis and biofilm formation was proposed [48].

With this knowledge of *C. testosteroni*, we explored other *Comamonas* genomes for *che* and *che-like* gene clusters (Figure 4). We found that nine of the 11 *Comamonas* genomes have *che* clusters, and they have consistent components and similar genetic organization to the *che* cluster of *C. testosteroni* CNB-1. We also found that six of the 11 *Comamonas* genomes have gene clusters similar to the *flm* cluster of *C. testossteroni* CNB-1, suggesting that coordination of chemotaxis and biofilm formation might occur also in other *Comamonas* species. Interestingly, we observed that some of the *che-like* clusters of *C. badia* and *C. granuli* have annotated *pilTs*, of which the production is a Tfp pilus assembly protein and can regulate twitching motility, and other components are consistent with the *flm* cluster. Some additional *che-like* clusters that have different genetic organization to *C. testosteroni* CNB-1 clusters were observed in *C. granuli* and *C. aquatica* genomes, respectively (Figure 4). So far, the functions of these additional *che-like* clusters are still unknown.

## 7. Summary and Perspective 

The knowledge of bacterial chemotaxis toward aromatic compounds is of fundamental importance to understanding how bacteria adapt and survive in environments, and is also important for the development of biosensors for the detection of aromatic compounds and to improve bioremediation of aromatic-polluted environments. As an emerging environmental model organism, not only the ability of *C. testosteroni* to degrade aromatic compounds, but also its chemotaxis, has attracted more and more attention. Studies with *C. testosteroni* have revealed that chemotaxis towards aromatic compounds can be triggered via sensing their intermediate metabolites of Kreb’s cycle [36,37], and also provided the first evidence that chemoreceptor MCP2901 binds directly to aromatic compounds [38]. Chemotaxis helps bacteria to find aromatic compounds, and the degradation of aromatic compounds can produce more chemoeffectors to attract bacteria. A positive feedback loop is generated from this process. In addition, studies with *C. testosteroni* has also revealed novel functions of *che-like* clusters and new chemotactic proteins [48].

Considering the diversity of aromatic compounds and of the putative bacterial chemoreceptors, there are more “unknowns” than “knowns”. For examples, how chemoreceptors coordinate themselves for chemotaxis and other behaviors, and how the signals generated from chemoreceptors can be transmitted downstream, should be the focus of future research. In addition, what the functions are of novel *che-like* gene clusters, such as clusters in *C. granuli* and *C. aquatic*, is also an interesting question. Answers to these questions will not only improve our understanding of chemotaxis with *C. testosteroni* and other *Comamonas* species, but will also provide more insight into our understanding of sense and transduction of signals among chemotactic microorganisms.

## Figures and Tables

**Table 1:** Summary of the chemotactic responses of *Comamonas* species to various organic compounds.

Strain	Potential Chemoeffector		Refs
*Comamonas* sp. strain JS765	2-nitrotoluene		[35]
*Comamonas testosteroni* strain CNB-1	benzoate	2-hydroxybenzoate	[36,37,38]
	3-hydroxybenzoate	4-hydroxybenzoate	
	2,6-dihydroxybenzoate	protocatechuate	
	vanillic acid	vanillin	
	gallic acid	gentisate	
	phenol	catechol	
	adipate	succinate	
	fumarate	pyruvate	
	citrate	malate	
	α-ketoglutarate	*cis*-aconitate	
	oxaloacetate	isocitrate	
*Comamonas testosteroni* ATCC 11996	1-dehydrotestosterone	pregnenolone	[39]
	17α-hydroxyprogesterone	androstanedione	
	11α-hydroxyprogesterone	testosterone	
	21α-hydroxyprogesterone	deoxycorticosterone	
	5-androsten-3β-17β-diol		
